# Hybrid clustering strategies for effective oversampling and undersampling in multiclass classification

**DOI:** 10.1038/s41598-024-84786-2

**Published:** 2025-01-27

**Authors:** Amirreza Salehi, Majid Khedmati

**Affiliations:** 1 Department of Industrial Engineering, Sharif University of Technology, Tehran, Iran; 2 Department of Industrial Engineering, Sharif University of Technology, Azadi Ave., Tehran, 1458889694 Iran

**Keywords:** Multiclass classification, Imbalanced data, Oversampling, Undersampling, Ensemble, Applied mathematics, Computational science, Computer science, Scientific data, Statistics

## Abstract

Multiclass imbalance is a challenging problem in real-world datasets, where certain classes may have a low number of samples because they correspond to rare occurrences. To address the challenge of multiclass imbalance, this paper introduces a novel hybrid cluster-based oversampling and undersampling (HCBOU) technique. By clustering and separating classes into majority and minority categories, this algorithm retains the most information during undersampling while generating efficient data in the minority class. The classification is carried out using one-vs-one and one-vs-all decomposition schemes. Extensive experimentation was carried out on 30 datasets to evaluate the proposed algorithm's performance. The results were subsequently compared with those of several state-of-the-art algorithms. Based on the results, the proposed algorithm outperforms the competing algorithms under different scenarios. Finally, The HCBOU algorithm demonstrated robust performance across varying class imbalance levels, highlighting its effectiveness in handling imbalanced datasets.

## Introduction

Classification is one of the data mining techniques used to predict the class label of instances based on their features. Although most research efforts concentrate on two-class classification, multiclass classification is one of the most challenging machine learning research topics^1^. There exist widespread applications of multiclass classification in machine learning where fraud detection^2^, disease diagnosis^3^, Sentiment analysis^4^, plant species recognition^5^, and image classification^6^are some examples of this problem. On the other hand, it is important to acknowledge that numerous real-world datasets exhibit a significant disparity in the number of instances across different classes^7^. This is known as the multiclass imbalance classification problem. Given this problem, the machine learning models may perform poorly for the minority class due to bias toward the majority class^8^. To deal with the imbalance issue in classification, several methods have been proposed in the literature, which can be classified into four categories. Algorithm-level approaches mitigate class imbalance by implementing modifications and enhancements to classification algorithms. Data-level approaches address the class imbalance by adjusting the dataset through techniques such as undersampling the majority class or oversampling the minority class to mitigate the effects of skewed class distribution on the learning process. Cost-sensitive approaches seek to minimize the total misclassification cost by integrating algorithm-level and data-level strategies. Finally, ensemble methods, such as bagging and boosting, combine multiple classifiers to improve predictive performance. However, in most cases, the ensemble algorithms are combined with data-level or algorithm-level techniques^9^.

Multiclass classification, involving more than two classes, presents significant challenges due to the increased complexity of decision boundaries. In contrast, building a classifier to distinguish only between two classes can be easier since the decision boundaries can be more straightforward. Multiclass classification problems can be addressed by decomposing them into binary subproblems using various techniques, such as one-versus-one (OVO) and one-versus-all (OVA). The OVO technique divides a multiclass problem into many binary problems, with each binary classifier learning to differentiate between a pair of classes. The final predicted class is determined by aggregating the outputs of the individual base classifiers. The OVA technique trains a binary classifier for each class to differentiate it from all other classes. When a base classifier yields a positive prediction, the corresponding class is assigned as the output^10^. Despite the existence of multiple methods proposed in the literature for addressing imbalanced data, there exist some drawbacks to these methods. In this regard,

- Existing balancing methods primarily address binary datasets, and fewer approaches have been proposed to handle multiclass decomposition schemes in real-world datasets.
- The utilization of oversampling techniques leads to an expansion in the dataset size, which poses challenges when applying learning operations to the dataset.
- Undersampling techniques, on the other hand, result in a significant loss of information due to the removal of samples.
- Oversampling techniques that involve duplicating samples can lead to overfitting on the training dataset, compromising the generalization capabilities of the model.
- The random nature of undersampling and oversampling techniques may not accurately represent the true features of the dataset, causing potential biases in data.
- Algorithm-level and ensemble algorithms, when applied independently, often exhibit inconsistent performance and are susceptible to the challenges posed by imbalanced datasets.
- Sampling from the majority classes in highly imbalanced datasets can result in model underfitting on majority classes, affecting the overall performance.
- Methods based on clustering often disregard the small number of samples present in each cluster, which can affect the effectiveness of these methods.
- Some methods for multiclass classification cannot be applied in different decomposition schemes, limiting their usability.

In this paper, we propose a novel hybrid cluster-based oversampling and undersampling (HCBOU) algorithm for classifying multiclass imbalanced datasets that combines two data-level techniques, including oversampling and undersampling, with K-means clustering. The proposed algorithm utilizes both OVO and OVA decomposition techniques for classification. The combined application of oversampling and undersampling techniques can effectively mitigate the risks of overfitting, which may arise from exclusive reliance on oversampling, and information loss, which can occur when undersampling is applied excessively. In this approach, the classes of datasets are categorized into two groups, including majority and minority, depending on the number of data instances. This categorization would result in multiple majority or minority classes. Then, the algorithm employs a clustering technique within each minority group to identify relevant clusters and generate more meaningful data. Furthermore, sampling is carried out in each majority class using a clustering-based approach, which minimizes the influence of noisy data and effectively reduces information loss. The objectives and contributions of this research study are summarized as follows:

- We propose a novel hybrid approach that combines undersampling, oversampling, and clustering techniques to address class imbalance. This algorithm effectively balances multiclass imbalanced datasets by combining the strengths of both data-level methods and ensemble learning. By leveraging K-means clustering, we ensure more meaningful data generation for minority classes while minimizing noise and information loss in majority classes.
- The effectiveness and reliability of HCBOU are thoroughly validated through extensive experiments conducted on 30 datasets, each with varying degrees of class imbalance. The proposed algorithm demonstrates superior performance compared to six state-of-the-art algorithms across multiple evaluation metrics, including precision, recall, and F1 score. This comprehensive assessment underlines the generalizability and consistency of HCBOU across a wide range of real-world imbalanced datasets.
- The proposed HCBOU algorithm employs both OVO and OVA decomposition schemes, ensuring its flexibility and robustness across different multiclass scenarios. This dual decomposition not only addresses the complexity of multiclass imbalanced classification but also enables more precise decision boundaries by simplifying complex multiclass problems into manageable binary sub-problems.

The remainder of the paper is organized as follows. Section “Literature review” provides a comprehensive review of existing research on classifying imbalanced multi-class datasets. The details of the proposed approach are provided in Sect.“The proposed HCBOU algorithm”. Section “Experiments” provides the experimental setup. The performance of the proposed approach is evaluated and compared to the competing algorithms in Sect. “Results”. Finally, Sect. :“Conclusions”. provides the conclusions and some future research recommendations.

## Literature review

As mentioned in the previous section, data balancing methods can be classified into four categories. In this section, a comprehensive literature review of research studies on data balancing methods is presented.

### Data-level methods

Data-level approaches address the class imbalance by adjusting the training dataset through techniques such as oversampling or undersampling. Oversampling involves producing more instances of the minority class by duplicating existing instances or generating new synthetic instances using methods like Synthetic Minority Oversampling Technique (SMOTE)^11^. On the other hand, undersampling entails eliminating a subset of instances from the majority class using methods like Tomek links^12^ or randomly deleting instances from the majority class. To name a few research works in the context of data-level methods, Gao et al.^13^ proposed the differential partition sampling ensemble method (DPSE) within the OVA framework. They classified all samples into safe examples, borderline examples, rare examples, and outliers. According to the distributional characteristics of the classes, random undersampling for safe examples and SMOTE for borderline and rare examples are then offered. Finally, they concluded that the proposed DPSE performs better than traditional techniques in OVA, OVO, and direct classification schemes. Krawczyk et al.^14^ introduced multiclass radial-based oversampling (MCRBO), a technique that employs potential functions to synthesize new instances. Finding regions with low mutual class distribution values guides synthetic instance generation. Li et al.^15^ presented a clustering-based technique for multiclass imbalanced problems. They initially split a multiclass dataset into several binary-class datasets and then, using spectral clustering divided the minority classes of the binary-class subsets into subspaces. After subspace identification, oversampling is performed tailored to the characteristics of each subspace. Liu et al.^16^ proposed a real-value negative selection detector-based oversampling method that modifies the traditional real-value negative selection technique to handle the multiclass imbalance problem. The loss of information of minority classes is reduced by minimizing the within-class imbalance. Neetha et al.^17^ proposed Borderline-DEMNET, addressing the class imbalance in Alzheimer's disease classification using Borderline-SMOTE, achieving high accuracy and outperforming previous multiclass classification models. Arafat et al.^18^ proposed a cluster-based undersampling approach to handle imbalanced multiclass classification problems. The suggested approach divides instances of the majority class into clusters, chooses the most useful instances within each cluster to generate several balanced datasets, and finally applies the random forest algorithm to the balanced datasets. Dai et al.^19^ proposed the Three-line Hybrid Positive Instance Augmentation (THPIA) algorithm, leveraging genetic principles to mix features of majority and minority classes, improving minority instance representation and reducing overfitting.

### Algorithm-level methods

The algorithm-level approach entails explicitly addressing the issue of class imbalance by applying some improvements in the existing learning algorithms or developing novel algorithms. In this regard, Chen et al.^20^proposed a double kernel-based class-specific broad learning system (DKCSBLS) to cope with multiclass imbalanced data. To put more emphasis on minority classes, the model includes class-specific penalty coefficients. Furthermore, a double kernel mapping approach aims to capture features with increased robustness. Vij and Arora^21^ proposed a deep transfer learning-based diagnostic system for multiclass diabetic retinopathy classification, using modified models (Inception V3, ResNet34, EfficientNet B0, VGG16, Xception) to enhance diagnosis accuracy, achieving 99.36% accuracy with balanced imbalanced data labels. Ding et al.^22^proposed a weighted online sequential extreme learning machine with kernels (WOS-ELMK) for both binary class and multiclass imbalance learning. The non-optimal hidden node problem related to random feature mapping in previous online sequential extreme learning machine (OS-ELM) approaches for imbalance learning is avoided by their proposed kernel mapping in WOS-ELMK. Ketu and Mishra^23^ proposed a scalable kernel-based SVM classification approach, which is based on the concept of the adjusting kernel scaling (AKS) approach to handle the multiclass imbalanced dataset. The chi-square test and weighting criteria have been used to evaluate the selection of the kernel function. Li et al.^24^ proposed a multiclass imbalance classification approach that incorporates a class imbalance ratio, density-based factor, and adaptive weighting mechanism, enhancing the distribution of weights for better handling imbalance issues. Dai et al.^25^ introduced a novel Schur decomposition class-overlap undersampling method (SDCU) to globally identify overlapping instances, enhancing classifier performance in imbalanced datasets by reducing overlap and noise. Han et al.^26^ proposed a global–local-based oversampling method (GLOS) that adjusts synthetic instance generation based on class-level and instance-level dispersion, improving the quality and relevance of the synthetic data for multiclass imbalance problems.

### Cost-sensitive methods

Cost-sensitive learning explicitly accounts for the misclassification costs while training a model and minimizes the expected cost of misclassification. For an imbalanced dataset, misclassifying the minority class is costlier than misclassifying the majority classes. Tapkan et al.^27^ presented a cost-sensitive approach that utilizes the Bees algorithm. The most significant advantage of this approach is its ability to handle binary and multiclass classification problems. Additionally, it can incorporate misclassification costs into the algorithm by generating neighboring solutions and evaluating the quality of the outcomes. Fernández et al.^28^ introduced a multiclass cost-sensitive classification technique called Boosting Adapted for Cost matrix (BAdaCost). It involves combining several cost-sensitive multiclass weak learners to create a powerful classification rule within the Boosting framework. Iranmehr et al.^29^ provided a constructive approach to improve the classifier about class imbalance by extending the basic SVM loss function. It can be demonstrated that the resulting classifier ensures Bayes consistency. Liu et al.^30^ developed a multiclass imbalanced and concept drift network traffic classification framework based on online active learning (MicFoal), addressing multiclass imbalance and concept drift in network traffic classification with online active learning, showing superior performance on eight real-world datasets. Yang et al.^31^introduced a deep reinforcement learning framework for handling multiclass imbalanced data in healthcare, enhancing minority class prediction by combining dueling and double deep Q-learning with custom reward functions. Mienye and Sun^32^ proposed robust cost-sensitive classifiers that efficiently predict medical diagnosis by modifying the objective functions of well-known algorithms, including logistic regression, decision tree, extreme gradient boosting, and random forest. Subsequently, the corresponding cost-sensitive algorithms for these models are developed. Unlike resampling techniques, this approach does not modify the original data distribution.

### Ensemble methods

As mentioned previously, the ensemble approaches apply multiple classifiers to improve learning accuracy. Combining ensemble algorithms with data-level approaches, the final performance of the model can be improved during the learning process. Liu et al.^33^ proposed an EasyEnsemble approach that deals with imbalanced datasets. In this approach, they created several subsets from the majority class where each subset is used to train a learner, and their outputs are combined to form a final prediction. Seiffert et al.^34^ proposed the RUSBoost algorithm, which combines undersampling and boosting approaches. Specifically, it utilizes random undersampling (RUS) to remove instances randomly from the majority class until the desired balance is achieved. Grina et al.^35^ proposed a re-sampling method based on belief function theory and ensemble learning, which assigns soft evidential labels to improve object selection for both undersampling and oversampling in multiclass imbalance. Wang et al.^36^ proposed an algorithm entitled SMOTEBagging that investigates the effects of diversity on each class. The model combines SMOTE with bagging to handle imbalanced classification problems. Hido et al.^37^ presented the Roughly Balanced Bagging method, which employs a novel sampling technique to enhance the original bagging algorithm for imbalanced datasets. Wang et al.^38^ presented a technique entitled AdaBoost.NC, as a combination of the multiclass classification AdaBoost algorithm with negative correlation learning. The initial weights given to the training examples in this approach are inversely correlated with the number of instances in each class. This approach enables the algorithm to better capture the complex relationships between different classes. Zhang et al.^39^ proposed an efficient framework entitled SMOTE + AdaBoost. To balance the dataset before AdaBoost, the SMOTE technique is used to generate synthetic majority classes. Chen et al.^40^ proposed a Balanced Random Forest that reduces bias toward the majority class and improves the accuracy of predictions on the minority class by randomly undersampling the majority class during the creation of each decision tree in the ensemble. Rodriguez et al.^41^ proposed the Random Balance strategy (RandBal) for creating classifier ensembles to deal with imbalanced two-class datasets. In RandBal, each base classifier is trained on a subset of data with a randomly assigned class distribution, regardless of an apriori distribution. Consequently, for each subset, one class will be undersampled while the other will be oversampled. Two approaches are available for handling multiclass problems: the first approach decomposes the problem into a series of binary problems, while the second approach, Multiple Random Balance (MultiRandBal), addresses all classes simultaneously. Dai et al.^42^ proposed a Heterogeneous Clustering Ensemble learning method for Multiple Class-overlap Detection (HCE-MCD) to address multiclass imbalance problems. The method uses a genetic algorithm to select and combine heterogeneous clustering techniques for effective overlap detection and utilizes majority voting for improved clustering results.

The proposed HCBOU algorithm distinguishes itself from existing methods by integrating clustering-based oversampling and undersampling techniques in a hybrid framework, ensuring more informed instance selection and generation. Unlike traditional oversampling or undersampling approaches, HCBOU leverages K-means clustering to improve data balance while minimizing noise and information loss. Additionally, HCBOU's dual decomposition using both OVO and OVA schemes ensures enhanced classification performance in multiclass imbalanced datasets, offering a more flexible and robust solution compared to the algorithm-level, cost-sensitive, and ensemble methods reviewed in the literature. The properties of the approaches stated in the literature review are summarized in Table 1.

**Table 1:** The state-of-the-art methods for imbalanced multiclass classification with their respective properties marked with a checkmark (✓).

Algorithm	Data level			Cost-sensitive	Algorithm level		Ensemble			Scheme			Clustering
	Undersampling	Oversampling					Bagging	Boosting		OVO	OVA		
Differential partition sampling ensemble (DPSE)	✔	✔					✔			✔	✔		
Multiclass radial-based oversampling (MCRBO)		✔								✔	✔		
OVA decomposition and spectral clustering		✔									✔		✔
Modified real-value negative selection oversampling		✔									✔		
Cluster-based Undersampling	✔						✔						✔
Double-kernel based class-specific broad learning system					✔								
Kernel based online learning					✔								
BEE-Miner				✔									
BAdaCost				✔				✔					
Cost-sensitive support vector machines				✔									
EasyEnsemble	✔							✔		✔			
RUSBoost	✔							✔		✔			
SMOTEBagging		✔					✔			✔			
Roughly Balanced Bagging	✔	✔					✔						
AdaBoost.NC	✔	✔						✔		✔	✔		
SMOTE + AdaBoost		✔						✔		✔			
Balanced Random Forest	✔						✔						
Scalable kernel‑based SVM classification					✔								
Cost-sensitive learning method				✔			✔	✔					
Deep Transfer Learning-based System					✔								
MicFoal				✔									
Deep Reinforcement Learning-based System				✔									
Borderline-DEMNET		✔											
Adaptive Weighting Mechanism					✔			✔					
THPIA Algorithm		✔			✔								
Belief Function & Ensemble	✔	✔						✔					
SDCU	✔				✔								
GLOS		✔			✔								
HCE-MCD							✔						✔
Random Balance	✔	✔					✔	✔		✔	✔		

## The proposed HCBOU algorithm

Despite the advancements in data-level, algorithm-level, and cost-sensitive approaches to handling class imbalance, existing methods still face significant limitations in effectively managing multiclass imbalanced datasets. Common oversampling techniques like SMOTE often lead to overfitting, while undersampling methods may cause loss of crucial information. Ensemble-based methods can mitigate these issues but often require extensive computational resources and are not always adaptable to complex class distributions. To address these challenges, we propose the HCBOU algorithm. HCBOU uniquely integrates clustering techniques to inform both oversampling and undersampling strategies, providing a more balanced and noise-free dataset while preserving minority class representation. This framework is specifically motivated by the need for improved classification performance and robustness in highly imbalanced, multiclass scenarios, where existing techniques either overgeneralize or introduce bias. In this section, we propose a novel algorithm called HCBOU for classifying multiclass imbalanced data. The details of the HCBOU algorithm are provided in the next subsection.

### HCBOU: Hybrid cluster-based oversampling and undersampling

**Fig. 1:**
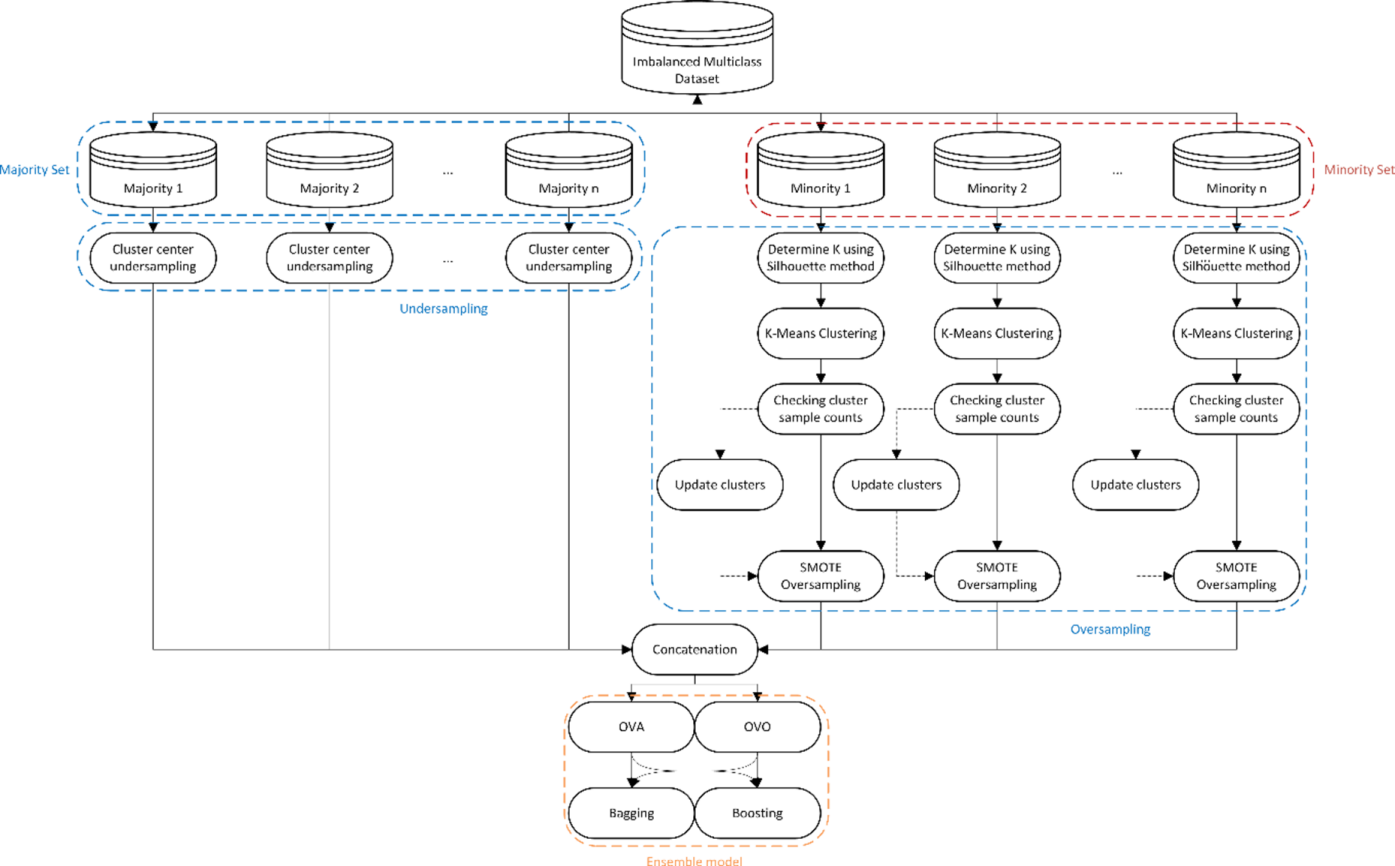
The process of the proposed HCBOU algorithm.

Multiclass imbalance poses a significant challenge in real-world datasets. Oversampling and undersampling methods have been proposed to tackle class imbalance, but a combination of these approaches can minimize information loss and overfitting risks. Despite the progress made in this area, achieving a balanced representation of all classes while maintaining data quality remains an ongoing issue, particularly when complex multiclass interactions are present. In this regard, maintaining the number of instances during data balancing is crucial for overall model simplicity. Furthermore, the impact of overlapping data points, which often leads to ambiguous decision boundaries, needs to be handled delicately to enhance model robustness. However, two key issues persist that need to be addressed: determining the data for new instance generation and selecting data from the majority class. This paper proposes a novel approach that combines oversampling, undersampling, and clustering methods to optimize sampling and data generation quality. Moreover, the algorithm integrates both OVO and OVA decomposition approaches to evaluate the performance across different decomposition schemes. This dual decomposition strategy enables a more granular evaluation of the model’s performance across various classes, providing insights into potential weaknesses in classification accuracy for minority classes. The proposed algorithm, illustrated in Fig. 1, demonstrates promising results in addressing the multiclass imbalance problem.

The notations used in this paper are represented in Table 2.

**Table 2:** The notations used in the proposed algorithm.

Notation	Size	Description
$N$	$1$	The number of instances in the training set
${N}_{{c}_{i}}$	1	The number of instances in class $i$
$M$	$1$	The number of attributes
$C$	$1$	The number of classes
$X$	$N\times M$	The set of features
$Y$	$N\times C$	The set of labels
α	$1$	The number of majority classes (the number of datasets in ${D}_{maj}$)
$\beta$	$1$	The number of minority classes (the number of datasets in ${D}_{min}$)
${D}_{i}$	${N}_{{c}_{i}}\times (M+1)$	The subset of training set containing the class $i$
${D}_{maj}$	$N_{{c_{i} }} \times \left( {M + 1} \right) \times {\upalpha }$	Majority set
${D}_{min}$	$N_{{c_{i} }} \times \left( {M + 1} \right) \times \beta$	Minority set
$S$	$1$	The number of instances of each class after balancing
${\Omega }_{i}$	${N}_{{c}_{i}}\times (M+1)$	The dataset containing cluster centres for dataset $i$
$O$	$1$	Optimal number of clusters for minority class clustering
${\widetilde{C}}_{i,j}$	$1$	The number of instances in cluster $i$ of dataset $j$
${nc}{\prime}$	$1$	The number of clusters with sufficient number of instances
${C}{\prime}$	${1\times nc}{\prime}$	The list of clusters with sufficient number of instances
*m*	$1$	Minimum number of instances in each cluster
${e}_{i,\dot{j}}$	$1$	The total absolute error (TAE) of the distance between instance $i$ and cluster $j$
$l{e}_{i}$	${1\times nc}{\prime}$	The list of TAE of the distance between instance $i$ and other clusters
$m{e}_{i}$	$1$	The cluster with the smallest TAE value for instance $i$
${W}_{i,j}$	$1$	The weight of cluster $i$ in the dataset $j$
${\widehat{S}}_{i,j}$	$1$	The number of instances generated in cluster $i$ of dataset $j$
$K$	1	The number of nearest neighbours in SMOTE
${D}{\prime}$	$(S\times C)\times (M+1)$	Balanced dataset
${\sigma }_{i}$	$1 \times M$	The centre of cluster $i$
${D}_{i,j,k}$	$1 \times M$	The sample $i$ from the cluster $j$ of dataset $k$

Initially, dataset $D$ is defined as a collection of sample-label pairs, represented as $\{(X, Y) | X\epsilon {\mathbb{R}}^{N\times M}, Y\epsilon {\mathbb{R}}^{N\times M}\},$ where $X$ represents the set of input features, and $Y$ represents the corresponding labels. To begin the process, the first step is to calculate the desired number of instances for each class, which is determined using Eq. (1), as shown below. Based on this, the dataset is divided into majority and minority groups using Eqs. (2)-(3):

1 $$S=\frac{N}{C}$$

2 $$\left\{D|class=i,({N}_{{c}_{i}}\ge S )\right\}\in {D}_{maj}$$

3 $$\left\{D|class=i,({N}_{{c}_{i}}<S )\right\}\in {D}_{min}$$

Based on these equations, if the subset containing class $i$ has more instances than $S$, it is placed in the majority class (${D}_{maj}$); otherwise, it is placed in the minority class (${D}_{min}$). This clear classification allows us to focus on addressing the imbalance more effectively.

**Fig. 2:**
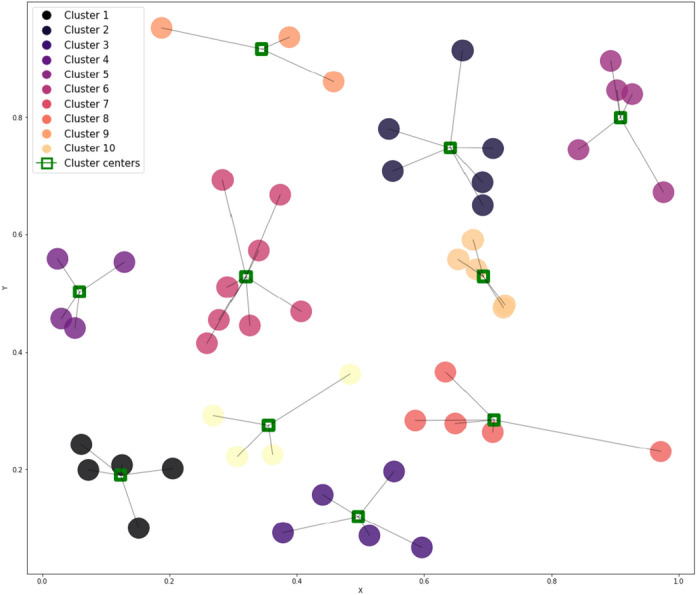
Illustration of using the cluster centres as replacements for the original data.

To handle the issue of imbalanced data and noise, clustering is applied in conjunction with undersampling. In this context, clustering groups data points based on their similarity, with the goal of maximizing intra-cluster similarity and minimizing inter-cluster similarity. Such clustering techniques help in forming coherent subgroups within the data, allowing for more targeted data manipulation. Instead of relying solely on undersampling, we employ a clustering strategy that ensures representative data points are retained. The centers of these clusters are used to represent the data, thereby reducing the risk of losing important information due to undersampling. This step is crucial because it helps to maintain data integrity while reducing the impact of noise and redundancy. Additionally, the use of clustering-based undersampling also allows for better handling of high-dimensional data, where traditional methods might struggle to capture the inherent structure of the dataset. However, it is important to note that sometimes this approach may not be effective if the clusters are not well-separated or if significant overlap exists between them. In those cases, adjustments are made to ensure proper separation between clusters. Figure 2 illustrates this approach, where data points are reduced from 50 to 10 while preserving the essential patterns.

**Fig. 3:**
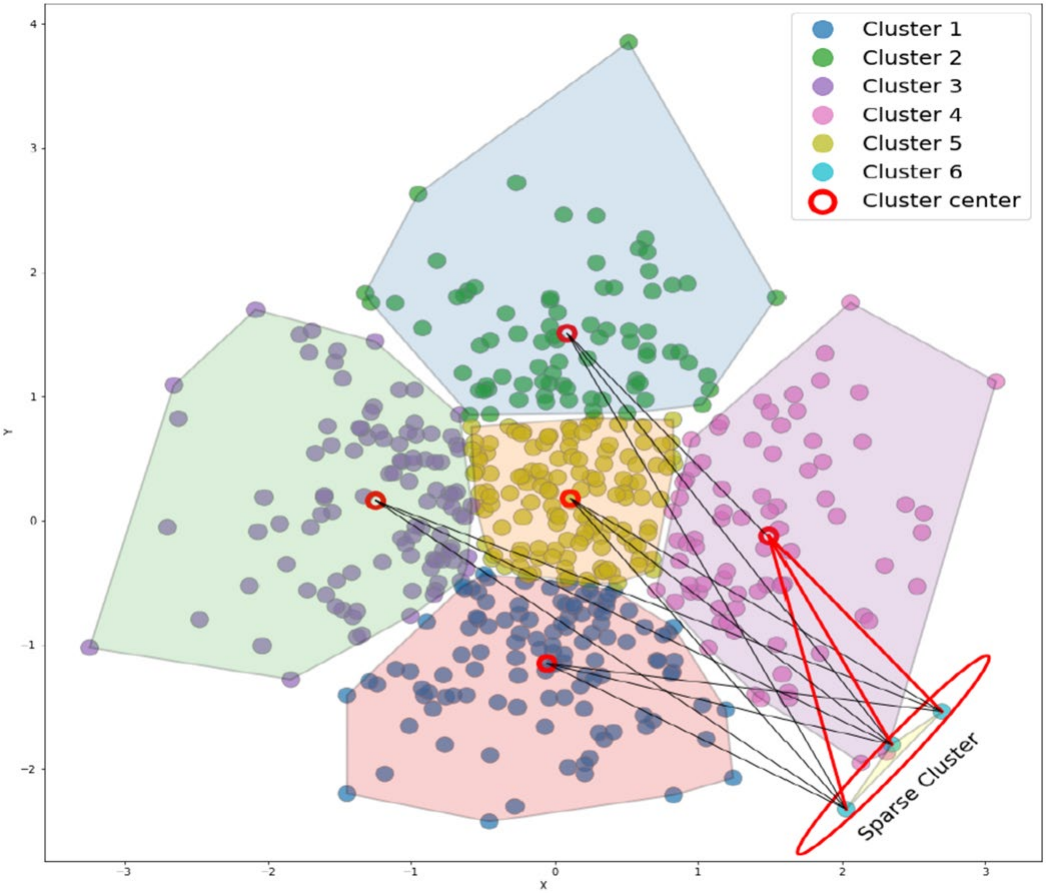
Illustration of data redistribution from sparse clusters.

Clustering is a fundamental step in our approach, as it allows us to effectively group data points, minimize intra-cluster variance, and optimize the sampling process, which is more critical than the specific clustering technique itself. Among various clustering methods, K-means is chosen because of its simplicity, efficiency, and suitability for large-scale datasets. The $$K$$-means clustering algorithm produces $$S$$ groups for each subset of ($${D}_{maj})$$. The centers of each cluster are then used as undersampled data. This clustering-based undersampling approach ensures that we retain representative data, even from large majority classes, without losing valuable information. For each subset of $${(D}_{\text{min}})$$, high-quality synthetic data are generated using a combination of $$K$$-means and oversampling. Here, we apply SMOTE in a localized manner within each cluster, which significantly reduces computation while improving the quality of synthetic data. Localized oversampling is particularly beneficial in cases where global oversampling would introduce noise or obscure meaningful patterns. This method ensures that the minority class is better represented, and the underlying data relationships are preserved. By focusing on localized cluster regions, this approach helps to mitigate the risk of generating redundant or irrelevant samples, which is a common issue in traditional oversampling techniques. By generating synthetic data for each cluster individually, we also avoid overfitting, a common risk when oversampling is applied to the entire dataset. In this regard, the well-known silhouette algorithm^43^ is used to find the best number of clusters $$(O)$$ for the $$K$$-means algorithm. Given that some clusters may contain a small number of instances after clustering, this could lead to the generation of low-quality data. To maintain consistency and avoid small, non-representative clusters, we establish a minimum threshold for the number of instances per cluster. To address this problem, at first, the clusters that have at least *m* instances are placed in $${C}{\prime}$$. The clusters with data points fewer than the threshold *m* are considered sparse clusters. Then, the data points from sparse clusters are redistributed to the nearest cluster from the list of $${C}{\prime}$$. The redistribution of sparse cluster data not only enhances the quality of generated samples but also helps in maintaining a smooth decision boundary. An illustration of data redistribution from sparse clusters is shown in Fig. 3.

Next, we calculate the appropriate amount of data to be generated for each cluster in (${D}_{min}$). The weight of the $j$^th^ cluster belonging to the $i$^th^ subgroup of ${(D}_{\text{min}})$ is denoted by ${W}_{j, {D}_{min}[i]}$. Once the weight is determined, it can be used to ascertain the adequate number of samples for generation in the $j$^th^ cluster belonging to the $i$^th^ subgroup of ${D}_{\text{min}}$, denoted by ${\widehat{S}}_{j, {D}_{min}[i]}$. The calculation of ${W}_{j, {D}_{min}[i]}$ and ${\widehat{S}}_{j, {D}_{min}[i]}$ is shown in Eqs. (4) and (5), respectively.

4 $${W}_{m,{D}_{min}[j]}=\frac{{\widetilde{C}}_{m,{D}_{min}[j]}}{\sum_{1}^{m}{\widetilde{C}}_{m,{D}_{min}[j]}}; m\in \left\{\text{1,2},\dots ,O\right\}$$

5 $${\widehat{S}}_{m,{D}_{min}[j]}={\lfloor W}_{m,{D}_{min}[j]}\times (S-{\widetilde{C}}_{m,{D}_{min}\left[j\right]})\rfloor ; m\in \left\{\text{1,2},\dots ,O\right\}$$

To address the disparity between the available data for each class and the desired number of samples per class, it is necessary to generate additional data. The weight of each cluster reflects the relative importance of the related cluster in the process of data augmentation. After determining the required size of data for generation, the SMOTE method can be used to generate the data. The dynamic nature of cluster-weighted data generation ensures that the synthetic data is well-distributed, reducing bias and enhancing the diversity of samples. One of the required parameters of the problem is $K$, which represents the number of neighbors considered for data generation. Finally, the balanced dataset is reconstructed, and predictive models are implemented using ensemble approaches. These ensemble models leverage OVO and OVA decomposition schemes, further enhancing the predictive power of the model by combining results from multiple binary classifiers. Algorithm 1 represents the pseudo-code of the proposed algorithm.

**Algorithm 1:**
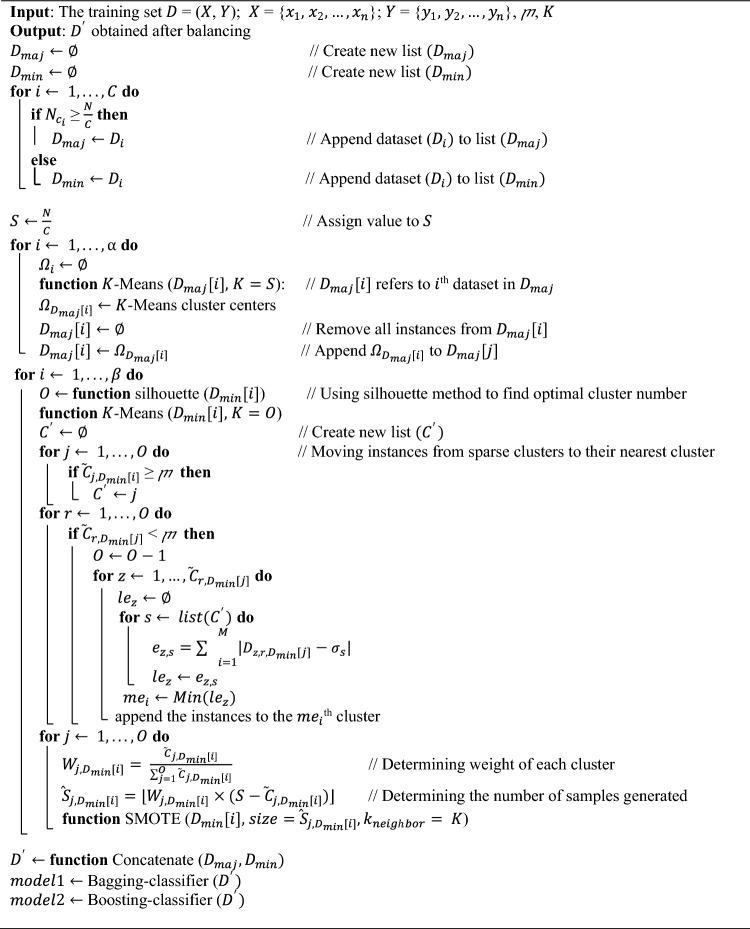
Hybrid cluster-based oversampling and undersampling for multiclass classification

## Experiments

This section evaluates the performance and validates the efficiency of our proposed approach in addressing multiclass imbalanced learning challenges. We will begin this section by providing a concise overview of the datasets, evaluation metrics, and comparison algorithms. Afterward, we will present the analysis and results of the experiments. We utilized the Python imbalanced-learn library^44^ to deal with imbalanced data.

### Datasets

The experiments involve 30 datasets with imbalanced classes, which were obtained from online repositories such as UCI, (https://archive.ics.uci.edu/datasets) KEEL (https://sci2s.ugr.es/keel/datasets.php) and OpenML. (https://www.openml.org/search?type=data&sort=runs&status=active) The imbalance ratio (IR), which is used to illustrate the degree of data imbalance, is defined as follows:

6 $$IR=\frac{\text{max}({N}_{{c}_{i}})}{\text{min}({N}_{{c}_{i}})}; i=1, 2,\dots , C$$

Table 3 provides an overview of these datasets. Each of the datasets contains at least three classes, and the imbalance ratio of the multiclass datasets ranges from 1.09 to 2160.

**Table 3:** 30 benchmark datasets are described along with their properties (#Ex: examples, #A: attributes, #Nu: numerical feature, #No: nominal feature, #C: classes).

Datasets	#Ex	IR	#A	#Nu	#No	#C	Class distribution
Arrhythmia	452	122.5	279	206	63	13	245, 50, 44, 25, 22, 15, 15, 13, 9, 5, 4, 3, 2
Autos	159	16.00	25	15	10	6	48, 46, 29, 20, 13, 3
Balance	625	5.87	4	4	0	3	288, 288, 49
Cardiotocography-10classes	2126	10.92	35	35	0	10	579, 384, 332, 252, 197, 107, 81, 72, 69, 53
Cardiotocography-3classes	2126	9.40	35	35	0	3	1655, 295, 176
Contraceptive	1473	1.88	9	6	3	3	629, 511, 333
Dermatology	366	5.60	34	34	0	6	112, 72, 61, 52, 49, 20
Ecoli	336	71.50	7	7	0	8	143, 77, 52, 35, 20, 5, 2, 2
Flags	194	15.00	28	2	26	8	60, 40, 36, 27, 15, 8, 4, 4
Glass	214	8.44	9	9	0	6	76, 70, 29, 17, 13, 9
Heart-cleveland	303	12.61	13	13	0	5	164, 55, 36, 35, 13
Heart-switzerland	123	9.60	12	12	0	5	48, 32, 30, 8, 5
Led7digit	500	1.54	7	0	7	10	57 57 53 52 52 51 49 47 45 37
Lenses	24	3.75	4	0	4	3	15, 5, 4
Lymphography	148	40.5	18	3	15	4	81, 61, 4, 2
Molec-biol-splice	3190	2.15	60	0	60	3	1655, 768, 767
New-thyroid	215	5.00	5	5	0	3	150, 35, 30
Nursery	12,960	2160	8	0	8	5	4320, 4266, 4044, 328, 2
Pageblocks	548	164.0	10	10	0	5	492, 33, 12, 8, 3
Penbased	1100	1.09	16	16	0	10	115, 115, 114, 114, 114, 106, 106, 106, 105, 105
Shuttle	2175	853.0	9	9	0	5	1706, 338, 123, 6, 2
Statlog-landsat	6435	2.44	36	36	0	6	1533, 1508, 1358, 707, 703, 626
Steel-plates	1941	12.23	27	27	0	7	673, 402, 391, 190, 158, 72, 55
Thyroid	720	39.17	21	6	15	3	666, 37, 17
Vehicle	846	1.09	18	18	0	4	218, 217, 212, 199
Vertebral-column-3classes	310	2.50	6	6	0	3	150, 100, 60
Wine	178	1.47	13	13	0	3	71, 59, 48
Winequality-red	1599	68.10	11	11	0	6	681, 638, 199, 53, 18, 10
Yeast	1484	92.60	8	8	0	10	463, 429, 244, 163, 51, 44, 35, 30, 20, 5
Zoo	101	10.25	16	0	16	7	41, 20, 13, 10, 8, 5, 4

### Evaluation metrics

Accuracy is a widely used metric to assess the effectiveness of a classification model. In the case of multiclass classification, accuracy can be defined as follows:

7 $$Accuracy = \frac{1}{N}\times \sum_{i=1}^{C}{TP}_{i}$$

where ${TP}_{i}$ stands for true positive of the $i$ ^th^ class, which means a positive sample is correctly identified as positive by the model. Also, $N$ is the total number of instances in the training set, and $C$ is the number of classes.

It should be noted that, in multiclass classification problems, the accuracy metric can be a misleading performance metric. Accuracy does not consider class distribution and favors the majority class, whereas a classifier that always predicts the majority class would still achieve a high accuracy. The F1-score^45^ is commonly used to evaluate multiclass classification models, where this metric is calculated per class and then averaged for an overall performance measure. This metric is a harmonic mean of precision and recall, and it is calculated for each class separately based on the following equation:

8 $$F1-scor{e}_{i}=\frac{\left(1+{\beta }^{2}\right){\times Precision}_{i}\times {Recall}_{i}}{{\beta }^{2}{\times Precision}_{i}+{Recall}_{i}}$$

Then, the arithmetic mean of all $F1-scor{e}_{i}$ becomes the model’s final F1-score, defined in Eq. (9):

9 $$F1=\frac{1}{C}\times \sum_{i=1}^{C}F1-scor{e}_{i}$$

where $\beta$ is utilized to balance the significance of precision and recall. In addition, recall and precision are defined as follows:

10 $$Precision_{i} = \frac{{TP_{i} }}{{TP_{i} + FP_{i} }}$$

11 $$Recall_{i} = \frac{{TP_{i} }}{{TP_{i} + FN_{i} }}$$

where ${FP}_{i}$ stands for false positive of the $i$ ^th^ class, which means a negative sample wrongly identified as positive by the model, and ${FN}_{i}$ stands for false negative of the $i$ ^th^ class, which means a positive sample wrongly identified as negative by the model.

Averaged-precision and averaged-recall^46^ are measures used in multiclass classification problems to calculate the average precision and recall values across all classes.

12 $$Averaged-precision=\frac{1}{C}\times \sum_{i=1}^{C}{Precision}_{i}$$

13 $$Averaged-recall=\frac{1}{C}\times \sum_{i=1}^{C}{Recall}_{i}$$

The G-mean score^47^ is another metric that can be used to assess the overall effectiveness of a multiclass classifier in a more balanced way. The G-mean in multiclass classification is calculated as the geometric mean of the recall scores for all the classes based on the following equation:

14 $$G-mean={\left(\prod_{i=1}^{C}{Recall}_{i}\right)}^\frac{1}{C}$$

The MAUC (Mean Area Under the Curve)^48^ is a measure that calculates the average AUC for all possible pairs of classes in a multiclass classification problem. This measure is defined in Eq. (15):

15 $$MAUC=\frac{1}{C(C-1)}\times \sum_{\underset{i\ne j}{i,j=1}}^{C}AUC(i,j)$$

where $AUC(i, j)$ represents the area under the curve that corresponds to the pair of classes $i$ and $j$.

### Parameter setting

In this study, we use a grid search technique to find the hyperparameter values that maximize the model's performance. Considering $K$ and *m* as two hyperparameters of the proposed model, the search space for these hyperparameters is $\left\{3, 4, 5\right\}$ and $\left\{3, 4, \dots , 15\right\}$, respectively. The performance of the proposed model is evaluated for all combinations of $K$ and values in the search space, and the hyperparameters that yield the best results are selected for the final model.

The performance of the proposed HCBOU is evaluated and compared to various multiclass imbalance learning techniques, including EasyEnsemble, RUSBoost, SMOTEBagging, Roughly Balanced Bagging, AdaBoost.NC and SMOTE + AdaBoost, all of which have demonstrated effective performance in handling imbalanced data. The parameter configurations of these approaches are represented in Table 4.

**Table 4:** Parameter settings for ensemble methods.

Method	Parameters
EasyEnsemble	estimator = AdaBoost; no. of estimators = 10; replacement = false
RUSBoost	no. of estimators = 50; replacement = false; learning rate = 1; estimator = Decision tree; estimator_depth $\in$ $\{\text{1,2},3\}$
Balanced Random Forest	no. of estimators = 100; replacement = false; estimator = Decision tree estimator_depth $\in$ $\{\text{1,2},3\}$ max _feature = $\sqrt{no. of feature}$
Roughly Balanced Bagging	no. of estimators = 10; estimator = Decision tree; estimator_depth $\in$ $\{\text{1,2},3\}$ bootstrap = true
AdaBoost.NC	no. of estimators = 100; learning rate = 1; estimator = Decision tree; estimator_depth $\{\text{1,2},3\}$
SMOTE + AdaBoost	estimator = AdaBoost; no. of estimators = 50; learning rate = 1; SMOTE_ k_neighbors $$\in$$$$\{\text{3,4},5\}$$
HCBOU	SMOTE_ k_neighbors $\in$ $\left\{\text{3,4},5\right\}$ $m$ $\left\{\text{3,4},\dots ,15\right\}$ Bagging: no. of estimators = 100; estimator = Decision tree; estimator_depth $\in$ $\left\{\text{1,2},3\right\}$ Boosting: no. of estimators = 50; estimator = Decision tree; estimator_depth $\in$ $\left\{\text{1,2},3\right\}$ learning rate = 1

Grid search is used to determine the optimal values of their parameters.

### Comparing imbalance learning approaches

The proposed method is compared with the methods EasyEnsemble, RUSBoost, Balanced Random Forest, Roughly Balanced Bagging, AdaBoost.NC and SMOTE + AdaBoost. The comparison is performed using both OVO and OVA classification schemes. Table 5 shows the proposed model and competing methods, along with their classification schemes. The proposed HCBOU is implemented using bagging and boosting approaches. The G-mean, F1-score, averaged precision, averaged recall, and MAUC metrics are used for comparisons across 30 datasets.

**Table 5:** Ensemble methods used along with their classification schemes.

	**Classification scheme**	
**Method**	**OVA**	**OVO**
EasyEnsemble	OVA-EE	OVO-EE
RUSBoost	OVA-RUS	OVO-RUS
Balanced Random Forest	OVA-BRF	OVO-BRF
Roughly Balanced Bagging	OVA-RBB	OVO-RBB
AdaBoost.NC	OVA-Ada.NC	OVO-Ada.NC
SMOTE + AdaBoost	OVA-S + Ada	OVO-S + Ada
HCBOU-Bagging	OVA-HCBOUBag	OVO-HCBOUBag
HCBOU-Boosting	OVA-HCBOUBoo	OVO-HCBOUBoo

## Results

The average rank of the methods in terms of six predefined metrics based on 30 datasets is presented in Table 6. The 95% confidence interval of the average ranks is also shown in this table ($\alpha =0.05$). The methods have been sorted from best to worst based on the average rank. Based on the results, it can be observed that the proposed method, in combination with bagging and OVA classification scheme (OVA-HCBOUBag), provides the best performance in terms of all evaluation metrics, except for the accuracy metric, where it ranks second. In addition, the proposed method in combination with bagging demonstrates better results compared to its combination with boosting, and the proposed method under the OVA classification scheme shows better performance compared to the OVO classification scheme.

**Table 6:** Average rank of performance metrics for the methods on 30 datasets.

Accuracy		Averaged-precision		Averaged-recall		F1		G-Mean		MAUC	
Algorithm	**Rank**	**Algorithm**	**Rank**	**Algorithm**	**Rank**	**Algorithm**	**Rank**	**Algorithm**	**Rank**	**Algorithm**	**Rank**
OVA-BRF	5.80 ± 1.5	*OVA-HCBOUBag*	5.90 ± 1.5	*OVA-HCBOUBag*	5.30 ± 1.5	*OVA-HCBOUBag*	5.40 ± 1.6	*OVA-HCBOUBag*	5.03 ± 1.6	*OVA-HCBOUBag*	5.10 ± 1.5
*OVA-HCBOUBag*	6.30 ± 1.5	*OVO-HCBOUBag*	6.13 ± 2.0	*OVO-HCBOUBag*	5.37 ± 1.9	*OVO-HCBOUBag*	5.53 ± 2.0	*OVO-HCBOUBag*	5.47 ± 1.9	*OVO-HCBOUBag*	5.37 ± 1.9
OVA-Ada.NC	6.47 ± 1.6	OVO-Ada.NC	7.40 ± 1.4	OVA-BRF	7.17 ± 1.5	OVA-BRF	7.00 ± 1.5	OVO-EE	6.87 ± 1.3	OVA-BRF	6.83 ± 1.5
OVO-S + Ada	6.63 ± 1.7	OVA-BRF	7.43 ± 1.5	OVO-EE	7.57 ± 1.6	OVO-S + Ada	7.53 ± 1.5	OVA-EE	7.53 ± 1.5	OVO-EE	7.63 ± 1.6
OVO-Ada.NC	7.03 ± 1.3	OVO-S + Ada	7.63 ± 1.6	OVA-S + Ada	7.87 ± 1.5	OVA-S + Ada	7.70 ± 1.6	*OVA-HCBOUBoo*	7.53 ± 1.7	*OVA-HCBOUBoo*	8.03 ± 1.5
OVA-S + Ada	7.07 ± 1.6	OVA-Ada.NC	7.90 ± 1.7	*OVA-HCBOUBoo*	7.93 ± 1.5	OVO-Ada.NC	7.90 ± 1.4	OVA-BRF	7.80 ± 1.5	OVO-S + Ada	8.20 ± 1.8
*OVO-HCBOUBag*	7.07 ± 1.8	*OVA-HCBOUBoo*	7.93 ± 1.7	OVO-S + Ada	8.10 ± 1.7	*OVA-HCBOUBoo*	8.17 ± 1.7	*OVO-HCBOUBoo*	7.90 ± 1.7	OVA-S + Ada	8.40 ± 1.7
OVO-EE	9.07 ± 1.5	OVA-S + Ada	8.00 ± 1.8	*OVO-HCBOUBoo*	8.27 ± 1.8	OVA-Ada.NC	8.50 ± 1.4	OVO-BRF	8.20 ± 1.4	*OVO-HCBOUBoo*	8.53 ± 1.8
OVA-RBB	9.20 ± 1.5	OVO-BRF	8.57 ± 1.6	OVO-BRF	8.77 ± 1.7	*OVO-HCBOUBoo*	8.63 ± 1.7	OVA-S + Ada	8.97 ± 1.7	OVO-BRF	8.70 ± 1.6
OVO-BRF	9.40 ± 1.7	*OVO-HCBOUBoo*	8.67 ± 1.8	OVO-Ada.NC	9.37 ± 1.4	OVO-BRF	8.80 ± 1.7	OVO-S + Ada	9.10 ± 2.0	OVO-Ada.NC	9.23 ± 1.5
*OVO-HCBOUBoo*	9.90 ± 1.5	OVO-EE	9.23 ± 1.7	OVA-Ada.NC	9.50 ± 1.4	OVO-EE	9.07 ± 1.6	OVO-RBB	9.53 ± 1.7	OVA-Ada.NC	9.33 ± 1.5
*OVA-HCBOUBoo*	10.00 ± 1.6	OVA-RBB	9.27 ± 1.3	OVA-EE	9.53 ± 1.9	OVA-RBB	9.77 ± 1.4	OVA-RUS	9.80 ± 1.7	OVA-EE	9.40 ± 1.8
OVO-RBB	10.37 ± 1.5	OVA-RUS	10.07 ± 1.8	OVO-RBB	10.03 ± 1.8	OVA-RUS	10.20 ± 1.9	OVA-RBB	9.93 ± 1.2	OVO-RUS	10.17 ± 1.6
OVA-RUS	10.43 ± 1.9	OVA-EE	10.37 ± 2.0	OVA-RUS	10.20 ± 2.0	OVO-RBB	10.23 ± 1.5	OVO-Ada.NC	10.60 ± 1.7	OVA-RUS	10.20 ± 1.9
OVA-EE	10.60 ± 2.0	OVO-RUS	10.70 ± 1.5	OVA-RBB	10.50 ± 1.4	OVO-RUS	10.70 ± 1.5	OVO-RUS	10.80 ± 1.6	OVO-RBB	10.40 ± 1.7
OVO-RUS	10.67 ± 1.5	OVO-RBB	10.80 ± 1.4	OVO-RUS	10.53 ± 1.6	OVA-EE	10.87 ± 2.0	OVA-Ada.NC	10.93 ± 1.6	OVA-RBB	10.47 ± 1.3

The proposed method in italic.

To evaluate the significance of the differences between the OVA-HCBOUBag (the proposed method with the best performance) and other methods, a Wilcoxon signed-rank test^49^ at the significance level of $\alpha =0.05$ is performed on six performance metrics where the results are presented in Table 7. The Wilcoxon signed-rank test is a non-parametric statistical test for comparing two methods and is appropriate when data violates the assumptions of normality or equal variances. The null hypothesis for the Wilcoxon signed-rank test states that there is no significant difference between the two related groups being compared. The statistical analysis showed that the proposed method significantly improved all metrics compared to the other methods (p-value < 0.05).

**Table 7:** Results of Wilcoxon signed-rank tests comparing the proposed OVA-HCBOUBag method with other methods applied on 30 datasets, where the ( =) sign denotes no significant difference between the compared techniques, while ( >) indicates that the proposed method outperforms the compared method.

Algorithm	Accuracy	Averaged-Precision	Averaged-recall	F1	G-Mean	MAUC
OVA-EE	9.45E-03 ( >)	7.43E-03 ( >)	1.23E-02 ( >)	2.31E-03 ( >)	6.96E-02 ( =)	8.38E-03 ( >)
OVO-EE	4.68E-02 ( >)	2.11E-02 ( >)	9.87E-02 ( =)	1.01E-02 ( >)	8.00E-02 ( =)	5.27E-02 ( =)
OVA-RBB	2.10E-02 ( >)	3.55E-03 ( >)	9.52E-05 ( >)	2.64E-04 ( >)	8.74E-05 ( >)	6.77E-05 ( >)
OVO-RBB	3.02E-03 ( >)	5.11E-04 ( >)	7.20E-04 ( >)	7.05E-04 ( >)	1.46E-03 ( >)	2.37E-04 ( >)
OVA-BRF	5.77E-01 ( =)	1.84E-01 ( =)	5.53E-02 ( =)	1.55E-01 ( =)	1.59E-02 ( >)	6.51E-02 ( =)
OVO-BRF	1.46E-02 ( >)	4.80E-02 ( >)	2.23E-02 ( >)	2.17E-02 ( >)	1.56E-02 ( >)	1.20E-02 ( >)
OVA-RUS	6.18E-03 ( >)	6.17E-03 ( >)	3.03E-03 ( >)	1.94E-03 ( >)	1.62E-03 ( >)	1.81E-03 ( >)
OVO-RUS	2.90E-03 ( >)	1.86E-03 ( >)	7.45E-04 ( >)	8.78E-04 ( >)	4.77E-04 ( >)	8.45E-04 ( >)
OVA-S + Ada	6.21E-01 ( =)	1.38E-01 ( =)	3.06E-02 ( >)	6.51E-02 ( =)	6.16E-03 ( >)	9.40E-03 ( >)
OVO-S + Ada	8.45E-01 ( =)	1.52E-01 ( =)	3.06E-02 ( >)	7.00E-02 ( =)	6.76E-03 ( >)	1.74E-02 ( >)
OVA-Ada.NC	8.53E-01 ( =)	4.79E-02 ( >)	5.77E-04 ( >)	5.97E-03 ( >)	1.51E-04 ( >)	5.85E-04 ( >)
OVO-Ada.NC	5.71E-01 ( =)	2.02E-01 ( =)	2.15E-03 ( >)	3.57E-02 ( >)	9.15E-04 ( >)	1.47E-03 ( >)

For a more comprehensive comparison between the proposed method and other competing methods, Table 8 shows the pair-wise performance of the methods against each other. In this table, each cell shows the number of superiorities of the methods shown in rows over those shown in columns. According to the results, the proposed method resulted in superior effectiveness in comparison to the competing methods in terms of all performance measures and in most of the datasets. Furthermore, the proposed algorithm has demonstrated better performance using the bagging approach under most scenarios.

**Table 8:** Pair-wise comparison of methods based on statistical significance across 30 datasets: Number of significant wins in (a) accuracy, (b) averaged-recall, (c) averaged-precision, (d) F1, (e) G-Mean, and (f) MAUC, metric for the row method over the column method.

(a)																
	OVA-HCBOUBag	OVO-HCBOUBag	OVO-HCBOUBoo	OVA-HCBOUBoo	OVO-RBB	OVA-RBB	OVO-S+Ada	OVA-Ada.NC	OVO-RUS	OVO-Ada.NC	OVA-BRF	OVO-EE	OVA-RUS	OVA-S+Ada	OVO-BRF	OVA-EE
OVA-HCBOUBag		20	26	26	20	20	13	15	24	14	14	22	20	15	21	21
OVO-HCBOUBag	20		23	23	20	18	15	12	24	14	13	20	20	16	20	20
OVO-HCBOUBoo	8	11		18	17	14	8	7	18	8	9	16	16	11	15	19
OVA-HCBOUBoo	9	10	16		14	13	9	10	18	9	8	17	17	9	12	17
OVO-RBB	10	10	15	16		12	8	6	16	7	7	15	14	8	14	18
OVA-RBB	10	13	17	18	21		10	7	20	8	8	17	17	10	16	18
OVO-S+Ada	17	16	23	21	23	21		16	24	18	16	21	24	22	21	25
OVA-Ada.NC	16	19	23	22	25	24	18		26	20	19	25	24	20	21	26
OVO-RUS	7	7	12	13	15	11	11	8		8	8	19	23	10	16	22
OVO-Ada.NC	17	17	23	23	24	23	19	18	27		18	25	25	17	23	27
OVA-BRF	18	18	22	23	24	24	17	18	25	18		24	23	19	26	26
OVO-EE	9	11	14	15	16	14	10	11	20	11	11		22	9	19	25
OVA-RUS	11	11	14	14	17	14	9	10	12	10	13	13		10	17	19
OVA-S+Ada	15	15	21	21	23	22	16	12	22	17	16	23	25		21	24
OVO-BRF	10	11	15	19	17	15	10	13	18	11	12	17	20	12		24
OVA-EE	10	11	11	14	13	14	7	9	12	8	10	10	15	8	13	

(b)																
	OVA-HCBOUBag	OVO-HCBOUBag	OVO-HCBOUBoo	OVA-HCBOUBoo	OVO-RBB	OVA-RBB	OVO-S+Ada	OVA-Ada.NC	OVO-RUS	OVO-Ada.NC	OVA-BRF	OVO-EE	OVA-RUS	OVA-S+Ada	OVO-BRF	OVA-EE
OVA-HCBOUBag		15	25	24	24	26	22	23	23	23	19	19	20	18	19	21
OVO-HCBOUBag	20		26	22	22	24	22	23	23	22	22	19	20	20	19	20
OVO-HCBOUBoo	6	6		12	18	18	17	21	20	18	15	16	17	17	17	18
OVA-HCBOUBoo	9	10	19		21	21	16	18	20	17	13	16	19	14	16	18
OVO-RBB	6	8	12	9		18	12	13	16	14	12	13	14	9	13	14
OVA-RBB	4	6	12	9	14		11	13	17	13	11	11	14	10	10	12
OVO-S+Ada	8	9	14	14	19	20		19	22	19	16	17	22	19	18	21
OVA-Ada.NC	8	8	9	13	18	18	14		20	19	13	17	21	15	16	20
OVO-RUS	8	8	10	11	15	14	11	14		10	9	11	21	11	14	19
OVO-Ada.NC	8	9	12	14	17	18	15	18	24		15	17	20	15	18	21
OVA-BRF	12	9	15	18	19	20	17	22	24	19		19	21	19	21	22
OVO-EE	12	12	14	15	18	20	14	18	23	18	14		22	15	20	26
OVA-RUS	11	11	13	12	17	17	9	12	13	13	13	11		13	14	15
OVA-S+Ada	12	11	14	16	22	21	16	17	20	19	15	17	19		21	22
OVO-BRF	12	12	13	15	18	21	13	18	19	15	15	14	20	12		21
OVA-EE	10	11	12	13	17	19	11	13	14	12	12	9	19	10	15	

(c)																
	OVA-HCBOUBag	OVO-HCBOUBag	OVO-HCBOUBoo	OVA-HCBOUBoo	OVO-RBB	OVA-RBB	OVO-S+Ada	OVA-Ada.NC	OVO-RUS	OVO-Ada.NC	OVA-BRF	OVO-EE	OVA-RUS	OVA-S+Ada	OVO-BRF	OVA-EE
OVA-HCBOUBag		16	24	21	26	23	18	19	23	18	19	20	20	18	18	20
OVO-HCBOUBag	18		20	21	23	21	20	18	23	18	20	20	20	18	20	20
OVO-HCBOUBoo	7	12		13	22	17	14	14	18	12	14	16	19	14	15	17
OVA-HCBOUBoo	12	11	18		22	18	15	17	20	13	15	17	20	14	16	19
OVO-RBB	4	7	8	8		13	11	9	15	7	10	16	14	9	13	16
OVA-RBB	7	9	13	12	19		13	13	18	8	13	17	14	13	16	19
OVO-S+Ada	12	11	17	15	20	18		17	23	17	17	19	22	19	19	23
OVA-Ada.NC	12	13	16	14	22	18	16		24	19	18	21	21	18	19	24
OVO-RUS	8	8	12	11	16	13	10	9		8	9	14	20	11	12	21
OVO-Ada.NC	13	13	18	18	24	23	17	17	26		20	24	24	17	19	25
OVA-BRF	12	11	16	16	21	18	15	17	24	14		23	20	17	21	24
OVO-EE	11	11	14	14	15	14	12	13	19	11	10		19	13	13	22
OVA-RUS	11	11	11	11	17	17	9	12	15	9	14	14		12	16	20
OVA-S+Ada	12	13	17	16	22	18	16	14	20	16	17	19	20		16	22
OVO-BRF	13	11	15	15	18	15	12	15	21	14	16	21	18	17		23
OVA-EE	11	11	13	12	15	12	8	9	13	8	10	13	15	10	12	

(d)																
	OVA-HCBOUBag	OVO-HCBOUBag	OVO-HCBOUBoo	OVA-HCBOUBoo	OVO-RBB	OVA-RBB	OVO-S+Ada	OVA-Ada.NC	OVO-RUS	OVO-Ada.NC	OVA-BRF	OVO-EE	OVA-RUS	OVA-S+Ada	OVO-BRF	OVA-EE
OVA-HCBOUBag		16	24	25	25	25	21	22	25	20	19	21	19	17	18	21
OVO-HCBOUBag	18		23	22	24	22	22	23	23	21	21	21	19	19	19	21
OVO-HCBOUBoo	7	9		13	18	19	13	19	20	13	14	15	17	15	16	17
OVA-HCBOUBoo	8	10	18		20	18	14	18	19	14	14	19	20	15	15	18
OVO-RBB	5	6	12	10		16	12	10	16	9	10	15	14	9	14	19
OVA-RBB	5	8	11	12	16		12	12	17	10	12	16	16	12	13	17
OVO-S+Ada	9	9	18	16	19	19		18	23	18	16	20	22	20	21	24
OVA-Ada.NC	9	8	11	13	21	19	15		25	18	16	20	20	18	19	24
OVO-RUS	6	8	10	12	15	14	10	8		8	9	13	21	10	13	23
OVO-Ada.NC	11	10	17	17	22	21	16	18	26		17	24	23	16	20	25
OVA-BRF	12	10	16	17	21	19	16	19	24	17		23	22	19	22	25
OVO-EE	10	10	15	12	16	15	11	13	20	11	10		20	11	17	25
OVA-RUS	12	12	13	11	17	15	9	13	13	10	12	13		10	15	19
OVA-S+Ada	13	12	16	15	22	19	15	14	21	17	15	21	22		21	24
OVO-BRF	13	12	14	16	17	18	10	15	20	13	14	17	19	12		26
OVA-EE	10	10	13	13	12	14	7	9	10	8	9	10	15	8	9	

(e)																
	OVA-HCBOUBag	OVO-HCBOUBag	OVO-HCBOUBoo	OVA-HCBOUBoo	OVO-RBB	OVA-RBB	OVO-S+Ada	OVA-Ada.NC	OVO-RUS	OVO-Ada.NC	OVA-BRF	OVO-EE	OVA-RUS	OVA-S+Ada	OVO-BRF	OVA-EE
OVA-HCBOUBag		17	25	23	24	25	22	24	23	23	21	21	21	18	20	22
OVO-HCBOUBag	20		25	21	22	23	22	24	23	22	22	20	20	20	19	20
OVO-HCBOUBoo	9	9		13	20	21	18	20	20	18	17	17	18	18	16	19
OVA-HCBOUBoo	11	12	19		22	22	16	18	19	17	15	17	20	17	16	20
OVO-RBB	8	10	12	9		23	16	17	19	17	17	16	17	14	16	18
OVA-RBB	7	9	11	9	17		13	16	20	16	15	15	17	13	13	16
OVO-S+Ada	10	11	15	15	21	25		23	23	23	20	20	24	23	20	24
OVA-Ada.NC	9	9	12	14	21	22	18		23	21	18	22	22	18	19	23
OVO-RUS	10	10	12	13	21	20	17	19		15	20	18	23	17	20	23
OVO-Ada.NC	10	11	14	15	20	22	18	23	26		21	20	23	19	21	23
OVA-BRF	12	11	15	17	22	24	19	25	24	20		22	23	21	22	25
OVO-EE	12	13	15	15	22	23	18	22	26	22	21		23	19	22	26
OVA-RUS	12	13	14	12	22	23	14	18	22	17	21	19		18	21	24
OVA-S+Ada	14	13	15	14	23	25	21	22	22	21	21	21	22		21	23
OVO-BRF	13	14	16	16	23	26	18	23	24	19	24	22	23	21		27
OVA-EE	11	13	13	12	22	24	15	18	22	17	20	19	21	17	20	

(f)																
	OVA-HCBOUBag	OVO-HCBOUBag	OVO-HCBOUBoo	OVA-HCBOUBoo	OVO-RBB	OVA-RBB	OVO-S+Ada	OVA-Ada.NC	OVO-RUS	OVO-Ada.NC	OVA-BRF	OVO-EE	OVA-RUS	OVA-S+Ada	OVO-BRF	OVA-EE
OVA-HCBOUBag		14	25	24	25	26	21	23	24	23	20	20	20	19	22	21
OVO-HCBOUBag	20		26	23	23	23	22	22	24	22	20	19	19	20	21	19
OVO-HCBOUBoo	6	6		12	18	19	17	19	19	17	13	15	17	17	15	18
OVA-HCBOUBoo	9	9	19		21	20	18	16	20	16	11	17	19	15	16	18
OVO-RBB	5	7	12	9		17	11	13	14	12	9	12	14	11	13	13
OVA-RBB	4	7	11	10	15		11	11	18	12	9	12	14	10	10	14
OVO-S+Ada	9	9	14	12	20	20		18	21	19	15	16	22	19	18	20
OVA-Ada.NC	8	9	11	15	18	20	15		18	19	12	15	20	16	16	19
OVO-RUS	7	7	11	11	17	13	12	15		12	12	12	20	14	13	20
OVO-Ada.NC	8	9	13	15	19	19	15	17	22		12	17	22	17	18	21
OVA-BRF	11	11	17	20	22	22	17	23	21	22		18	21	20	21	21
OVO-EE	11	12	15	14	19	19	15	18	21	18	15		23	16	18	25
OVA-RUS	11	12	13	12	17	17	9	13	14	11	13	10		13	14	15
OVA-S+Ada	11	11	14	15	20	21	16	16	17	17	14	16	19		19	20
OVO-BRF	9	10	15	15	18	21	13	18	20	15	15	16	20	14		20
OVA-EE	10	12	12	13	18	17	11	14	13	12	13	10	19	12	15	

Table 9 presents a comparison of the performance of OVO and OVA schemes across various ensemble methods and evaluation metrics. The results indicate that the performance of the OVO is generally better than the OVA. This is justifiable due to the higher number of models in the OVO scheme. However, in some cases, it can lead to overfitting and inferior performance compared to the OVA scheme. Based on the evaluation of 30 datasets and different performance measures, it has been observed that HCBOU-Bagging demonstrates superior performance when employed in conjunction with the OVO scheme, whereas HCBOU-Boosting yields better results when utilizing the OVA scheme.

**Table 9:** Comparing OVA and OVO for various ensemble techniques and evaluation measures.

	**Ada.NC**			**BRF**			**EE**			**HCBOUBoo**			**HCBOUBag**			**RUS**			**RBB**			**S + Ada**			**SUM**	
	**OVA**	**OVO**		**OVA**	**OVO**		**OVA**	**OVO**		**OVA**	**OVO**		**OVA**	**OVO**		**OVA**	**OVO**		**OVA**	**OVO**		**OVA**	**OVO**		**OVA**	**OVO**
**Accuracy**	12	10		18	4		5	20		12	14		10	10		7	18		18	9		8	14		90	99
**Averaged-precision**	13	11		14	9		8	17		17	12		12	14		10	15		17	11		11	14		102	103
**Averaged-recall**	12	11		15	9		4	21		18	11		10	15		9	17		12	16		11	14		91	114
**F1**	12	12		16	8		5	20		17	12		12	14		9	17		14	14		10	15		95	112
**G-mean**	7	9		6	8		4	11		17	11		10	13		7	8		7	13		7	9		65	82
**MAUC**	13	11		15	9		5	20		18	11		10	16		10	16		13	15		11	14		95	112
**SUM**	69	64		84	47		31	109		99	71		64	82		52	91		81	78		58	80			

**Fig. 4:**
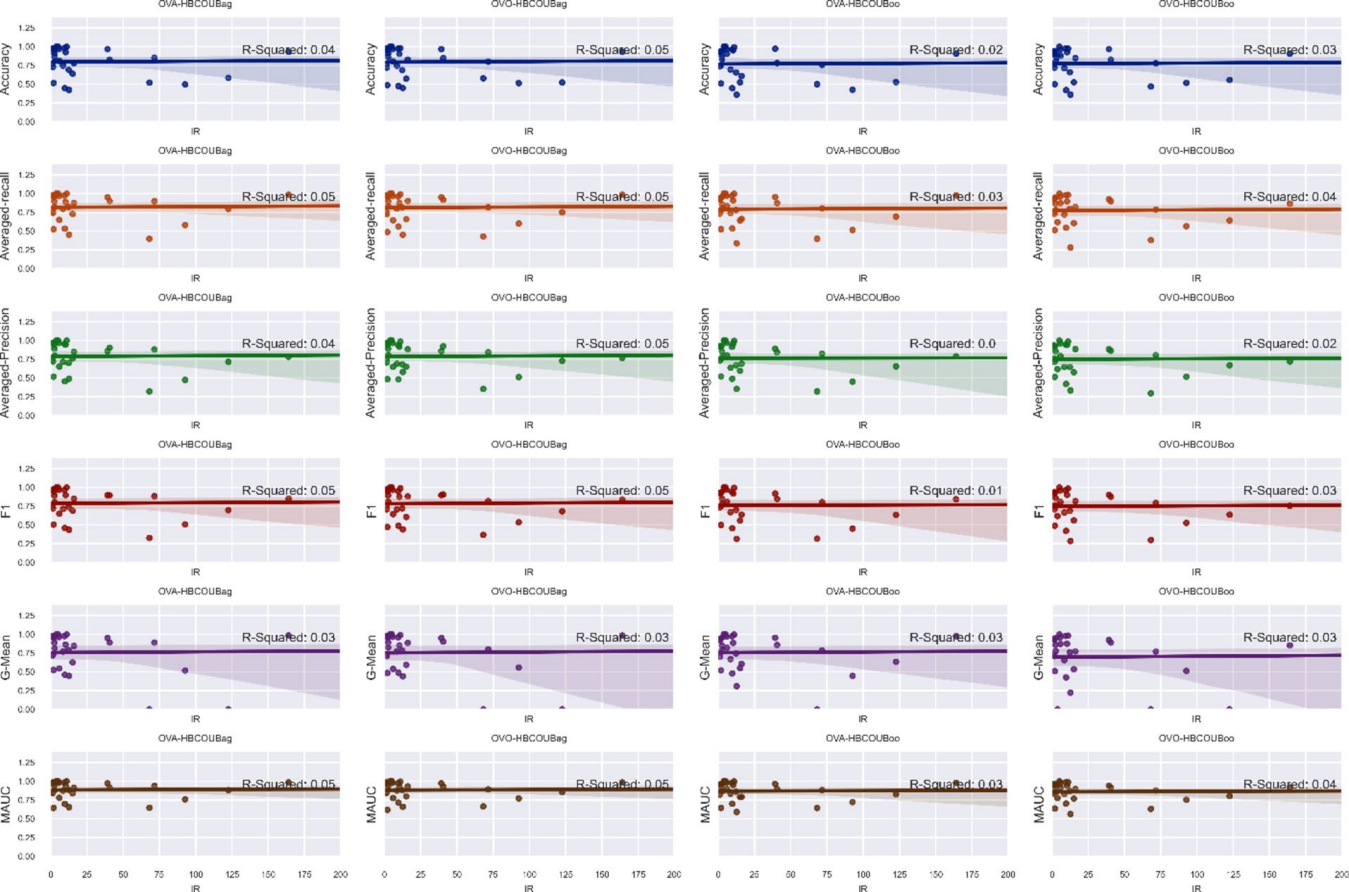
Analyzing the correlation between the imbalance ratio (IR) and performance measures.

Finally, Fig. 4 illustrates the relationship between the imbalance ratio (IR) and performance metrics using scatter plots and regression analysis. This plot can offer valuable insights into how the imbalance ratio affects performance metrics and can help identify any trends or patterns within the data. As the degree of class imbalance increases, the performance metrics remain relatively stable, suggesting that the method is consistently effective across various ratios of imbalance. However, some dataset performances may deviate from the fitted line due to internal variations within the data.

## Conclusions

Addressing class imbalance is a critical challenge in machine learning, particularly in domains such as healthcare, fraud detection, and text classification. While binary imbalanced classification has been extensively researched, multiclass imbalanced classification presents more intricate challenges due to the varying decision boundaries and complexities inherent in multiple classes. Despite substantial efforts in this area, current solutions often struggle with issues like overfitting during oversampling and information loss during undersampling. This study advances the field by introducing the Hybrid Cluster-Based Oversampling and Undersampling (HCBOU) algorithm, which effectively integrates clustering with data-level techniques to overcome these limitations. The HCBOU algorithm offers a novel approach to multiclass imbalanced classification by employing clustering to inform the sampling process, ensuring both the preservation of class structure and the generation of meaningful synthetic instances. This hybrid method improves upon existing techniques by addressing the delicate balance between reducing redundancy in majority classes and generating relevant data for minority classes, all while mitigating common issues like overfitting and class distortion. Furthermore, by leveraging one-vs-one (OVO) and one-vs-all (OVA) decomposition strategies, HCBOU enhances classification performance across diverse datasets, making it a versatile and powerful tool for real-world applications. The HCBOU algorithm first identifies class imbalances and applies clustering to divide majority and minority classes into coherent groups. It then performs undersampling on the majority classes to eliminate redundancy and oversampling on the minority classes to improve representation. The OVO and OVA decomposition techniques are employed to transform the multiclass problem into a series of binary tasks, which further enhances the algorithm’s precision. Experimental results across 30 diverse datasets demonstrate that HCBOU consistently outperforms six state-of-the-art algorithms, with significant improvements in precision, recall, and F1 scores. These findings confirm that the clustering-based approach significantly enhances data balance while maintaining the integrity of class relationships. The HCBOU algorithm holds significant potential for practical applications where minority class prediction is critical, such as medical diagnosis, fraud detection, and resource management. By improving the balance between minority and majority classes without compromising data quality, HCBOU enables better generalization in machine learning models. Its consistent performance across varied datasets underscores its robustness, making it a valuable contribution to both academic research and industry practices.

While the HCBOU algorithm demonstrates significant improvements in handling multiclass imbalanced classification, certain aspects could benefit from further refinement. The reliance on clustering methods, while effective, may increase computational demands in particularly large datasets, although this can be mitigated by optimizing parameters and using efficient clustering algorithms. Moreover, the choice of clustering technique can influence performance, but the flexibility of the HCBOU framework allows for adaptation based on the specific characteristics of the dataset at hand.

Future research should focus on optimizing HCBOU for large-scale and high-dimensional datasets, potentially by integrating dimensionality reduction techniques to alleviate computational burdens. Experimenting with alternative clustering methods, such as hierarchical or density-based clustering, could enhance the adaptability of the algorithm. There is also scope for developing an adaptive version of HCBOU that dynamically adjusts its sampling strategy based on evolving data characteristics. Finally, integrating cost-sensitive learning approaches could further improve the handling of multiclass imbalances, particularly in time-sensitive or real-time applications.

## Data Availability

The datasets generated and/or analysed during the current study are available in the [OpenML] repository, [https://www.openml.org/search?type = data&sort = runs&status = active], [Knowledge Extraction Evolutionary Learning] repository, [https://sci2s.ugr.es/keel/datasets.php] and [UC Irvine Machine Learning] repository, [https://archive.ics.uci.edu/datasets].

## Code availability

The code used in this study has been made publicly available to enhance reproducibility and verifiability of the research. It can be accessed at the following GitHub repository: https://github.com/Amir27Salehi/HCBOU.
